# Fibroblasts modulate epithelial cell behavior within the proliferative niche and differentiated cell zone within a human colonic crypt model

**DOI:** 10.3389/fbioe.2024.1506976

**Published:** 2024-12-16

**Authors:** Angelo Massaro, Cecilia Villegas Novoa, Yuli Wang, Nancy L. Allbritton

**Affiliations:** Department of Bioengineering, University of Washington, Seattle, WA, United States

**Keywords:** colon crypt, epithelial cells, microphysiological system, organ-on-chip, large intestine, pericryptal fibroblasts

## Abstract

Colonic epithelium is situated above a layer of fibroblasts that provide supportive factors for stem cells at the crypt base and promote differentiation of cells in the upper crypt and luminal surface. To study the fibroblast-epithelial cell interactions, an *in vitro* crypt model was formed on a shaped collagen scaffold with primary epithelial cells growing above a layer of primary colonic fibroblasts. The crypts possessed a basal stem cell niche populated with proliferative cells and a differentiated, nondividing cell zone at the luminal crypt end. The presence of fibroblasts enhanced cell differentiation and accelerated the rate at which a high resistance epithelial cell layer formed relative to cultures without fibroblasts. The fibroblasts modulated cell proliferation within crypts increasing the number of crypts populated with proliferative cells but decreasing the total number of proliferative cells in each crypt. Bulk-RNA sequencing revealed 41 genes that were significantly upregulated and 190 genes that were significantly downregulated in cocultured epithelium relative to epithelium cultured without fibroblasts. This epithelium-fibroblast crypt model suggests bidirectional communication between the two cell types and has the potential to serve as a model to investigate fibroblast-epithelial cell interactions in health and disease.

## 1 Introduction

The large intestine (colon) is a tubular organ of the gastrointestinal tract that absorbs water and nutrients while processing waste (Azzouz and Sharma, 2021). The colon is organized into concentrically stratified regions with the innermost layer consisting of epithelial cells that act as a high-resistance barrier to luminal contents (Peterson and Artis, 2014). The luminal epithelial surface is lined with an array of 100 µm wide, 500 µm deep invaginations called the crypts of Lieberkühn (Lieberkühn, 1760; Clevers, 2013). The base of the crypts provides a microenvironment supportive of long-lived stem cells enabling continuous tissue regeneration (Clevers, 2013). The stem cells proliferate rapidly, and their progeny migrate up the crypt long axis toward the luminal surface making fate decisions to become terminally differentiated epithelial cells covering the luminal intestinal surface. This orderly and continuous epithelial cell flow from the stem cell niche to the differentiated cell zone is due in part to an underlying layer of pericryptal fibroblasts which provide distinct physical and chemical cues to the intestinal epithelium based on location within a crypt (Roulis and Flavell, 2016). Rich bidirectional communication between fibroblasts and epithelium occurs via secreted factors, direct cell-to-cell contact, and mechanical cues from the fibroblast-deposited extracellular matrix (ECM) (Kabiri et al., 2014; Aoki et al., 2016; Roulis and Flavell, 2016; Valenta et al., 2016; Stzepourginski et al., 2017). To support and maintain the stem cells, fibroblasts near the crypt base secrete Wnt and R-spondin as well as bone morphogenetic protein (BMP) antagonists such as Gremlin and Noggin (Kabiri et al., 2014; Roulis and Flavell, 2016; Valenta et al., 2016; Chalkidi et al., 2022). In contrast, fibroblasts closer to the lumen direct cell differentiation through Notch signaling, secreted BMPs and non-canonical Wnt ligands (Brugger et al., 2020). Fibroblasts are also thought to lay down a spatial gradient of collagen, fibronectin, and laminin to support the epithelial cells along the crypt length (Roulis and Flavell, 2016; Wang et al., 2018a). Alterations in fibroblast signaling are now known to play critical roles in driving epithelial cell dysfunction (Li et al., 2022; Gauthier et al., 2023). For example, fibroblasts can become senescent secreting inappropriate levels of growth factors fueling the development of mutations in the stem cells increasing the likelihood of cancer initiation (Dang et al., 2021).

Most *in vitro* models of the intestinal mucosa have focused solely on the epithelium; however, increasingly fibroblasts are integrated into coculture models to investigate the interactions between the two cell types (Berschneider and Powell, 1992; Vachon et al., 1993; Viney et al., 2009; Lahar et al., 2011; Lei et al., 2014; Gregorio et al., 2018; Kinchen et al., 2018; Guo et al., 2019; Brugger et al., 2020; Nikolaev et al., 2020; Darling et al., 2020; Verhulsel et al., 2021; Macedo et al., 2021; Rudolph et al., 2022; Jasso et al., 2022). Fibroblasts cultured at a distance from the epithelial cells but sharing a common medium have permitted investigation of paracrine signaling (Sato et al., 2009; Miyoshi and Stappenbeck, 2013; Guo et al., 2019; VanDussen et al., 2019). Crucially, this work has enabled long-term *in vitro* culture of intestinal epithelial cells and identified secreted factors playing a role in fibroblast-epithelial cell interactions. Yet these models fail to capture the impact of direct cell-cell communication occurring *in vivo*. Multi-layered planar coculture models have been used to mimic pericryptal fibroblasts and examine direct cell-cell interactions (Darling et al., 2020; Macedo et al., 2021). These models have revealed how direct contact between cells influences tissue barrier function; however, these planar systems do not possess the complex 3D architecture or cell compartmentalization of the crypt. Epithelial organoids have been cultured within a fibroblast-laden hydrogel to mimic the fibroblast-filled stroma of the *in vivo* intestine (Lei et al., 2014). These methods revealed that a stroma containing fibroblasts supported the formation of a budding small intestine organoid. Nonetheless, these models did not mimic the pericryptal positioning of fibroblasts or support direct epithelial cell-fibroblast contacts. Microengineered intestine-on-chip systems with accurate crypt architecture and epithelial cell compartmentalization have been developed but to date these physiologically accurate systems do not fully incorporate pericryptal fibroblasts (Wang et al., 2017a; Wang et al., 2018b; Nikolaev et al., 2020; Verhulsel et al., 2021; Rudolph et al., 2022). Given that surface topography and curvature, direct cell-cell interactions, and cell compartmentalization are known to play critical roles in the physiology of the intestine, an improved understanding of the fibroblast-epithelial cell partnership awaits an architectural accurate model of the colonic epithelium with its pericryptal fibroblasts (Howard et al., 2021; Pentinmikko et al., 2022).

In this work, a 2D model and a micro-engineered 3D model was developed to enable colonic fibroblast-epithelial coculture. The planar or 2D coculture model was created for fast optimization of the coculture conditions with assessment of cell viability and surface coverage. This simple model utilized primary epithelial cells and a tissue-cultured fibroblast cell line CCD18-Co derived from normal human colon. Scaffolding and culture conditions were optimized for the epithelial cells while maintaining the wellbeing of the fibroblasts. The 3D model possessed *in vitro* crypts comprised of primary human colonic epithelial cells and underlying primary pericryptal fibroblasts isolated from normal colonic stroma. The optimized planar conditions were applied to the 3D model or array of crypts with underlying fibroblasts. Fibroblasts and epithelial cells (alone or in coculture) were assessed for their ability to proliferate under different conditions as well as their ability to express cell-type specific markers. Epithelial cell function, for example, creation of a high resistance barrier, was evaluated over time. The impact of the fibroblasts on epithelial cell crypt formation and cell compartmentalization was also evaluated. The impact on stem cell maintenance and proliferation was quantified followed by RNA expression analysis to investigate how the fibroblasts modified epithelial cell physiology. This system will enable an improved understanding of the interconnected relationship between epithelial cells and their partner cells, the pericryptal fibroblasts.

## 2 Materials and methods

### 2.1 Fabrication of crosslinked collagen scaffolds

2D and 3D collagen scaffolds were constructed on the surface of modified 12-well culture inserts (Corning, cat. No. 354236) according to Hinman et al. (2021). Briefly, the factory-supplied membrane was replaced with a permeable membrane (Millipore-Sigma, cat. No. BGCM00010) affixed with a biocompatible adhesive (3M, cat. No. 1504XL). A diffusion window was created by affixing another non-permeable polycarbonate film (McMaster-Carr, cat. No. 8689K44) with a 3 mm hole cut out. The optimal size of diffusion window was 3 mm diameter which enabled the luminal and basal reservoirs to act as an infinite source and sink and form a stable linear gradient across the long (Z) axis of the crypt over 24 h (Wang et al., 2017b). Replenishment of the media in the luminal and basal reservoirs every 24 h enabled formation of long term gradients of molecules at dissimilar concentrations in the luminal and basal reservoirs, *e.g.*, WRN. For the cross-linked collagen scaffold, N-(3-dimethylaminopropyl)-N′-ethylcarbodiimide hydrochloride (EDC) and N-hydroxysuccinimide (NHS) were mixed with rat tail type I collagen (Corning, cat. No. 354236) and this mixture, while still liquid, was added to the culture insert atop the diffusion window and then pressed into form by a flat or shaped polydimethylsiloxane (PDMS) stamp. The shaped stamp used for 3D crypt scaffolds contained an array of 150 µm wide by 600 µm tall posts and it was fabricated according to protocols from Hinman et al. (2021). To accommodate fibroblast-epithelium coculture, a photomask with larger hole diameters (150 µm) was used for mold construction and an additional layer of photoresist was added during spin coating to increase crypt height (1,500 rpm for 30 s with an acceleration of 300 rpm/s). Prior to cell plating, the collagen scaffolds were surface treated by overnight incubation with 10 μg/mL type 1 human collagen (Advanced BioMatrix, cat. No. 5007-20ML) in phosphate buffered saline (PBS).

### 2.2 Cell culture

All methods were carried out in accordance with relevant guidelines and regulations stipulated by the National Institutes of Health. The experimental protocols were approved by the University of Washington. The cell line used in this work was derived from de-identified cadaveric transplant donor intestines made available through an NIH-funded biobank. The original tissues to create this biobank were obtained from a federally designated organ procurement organization. Transplant donors were consented and de-identified by the procurement organization.

Human colon epithelial stem cells from a transverse colon tissue sample (male, 23 years old, RRID: CVCL_ZR41) were cultured atop a neutralized collagen slab within maintenance media (MM, Supplementary Table S1) according to previously described methods (Wang et al., 2017a). Fibroblasts were expanded within a tissue culture flask in fibroblast medium (FM) containing Dulbecco’s Modified Eagle Medium (DMEM, Thermo Fisher, cat. No. 11995065) supplemented with 10% heat-inactivated fetal bovine serum, 100 U/mL penicillin, and 100 μg/mL streptomycin (Supplementary Table S1). For planar experiments CCD18-Co fibroblasts (colon, female, 2.5 months old, ATCC: CRL-1459) were used, and for 3D crypt experiments primary fibroblasts isolated from descending colon (male, 12 years old, RRID: CVCL_D6WE) were used. Primary fibroblasts were obtained from human tissue by isolating adherent cells from the transverse colon as described previously (Khalil et al., 2013).

For seeding within molded scaffolds, fibroblasts were added to the luminal side of well-inserts in 1 mL FM at a concentration of 3 × 10^5^ – 5 × 10^5^ cells/mL for both planar and 3D crypt scaffolds. Then FM was replenished every day for up to 7 days, or until confluent. For subsequent epithelium addition, epithelial stem cells grown on 6-well plate above soft neutralized collagen were isolated and dissociated according to previous protocol using collagenase type 4 (Worthington Biochemical Corporation, cat. No. LS004189) and TrypLE express enzyme (Thermo Fisher, cat. No. 12605028) (Hinman et al., 2021). Epithelial cells were then added directly to the scaffolds for monoculture, or directly on top of fibroblasts for coculture, at a ratio of 1:3 (1 well from 6-well maintenance plate to 3 well-inserts). N.b. It was critical that epithelial cells were cultured in neutralized collagen maintenance wells for only 3–4 days before isolation and sufficiently dissociated prior to addition to maximize the stem cell population upon initial seeding. In planar culture, the cells were then grown in expansion media (EM, modified from Hinman et al. See Supplementary Table S1) with or without exogenously supplied Wnt, R-spondin, and Noggin (WRN) from L-WRN conditioned media for 12 days. In 3D crypts, cells were grown in EM + WRN for 8 days (replenished daily) until a confluent layer was observed on the luminal, inter-crypt surface and all crypts appeared full and darker in brightfield imaging. Polarization was then executed by replacing the medium in the basal reservoir with stem medium (SM, Supplementary Table S1) and luminal medium was replaced with differentiation medium (DM, Supplementary Table S1). After 4 days of polarization (with media replenished daily), the luminal surface developed a full and wavy layer of differentiated epithelium as observed by transmitted light microscopy. The samples were then fixed and stained for endpoint analysis.

### 2.3 Measuring barrier function

An EVOM2 Epithelial Voltohmmeter (World Precision Instruments) with a chopstick electrode was used to measure transepithelial electrical resistance (TEER) in planar samples. The background resistance was measured in cell-free, molded collagen scaffolds, and the effective surface area was considered 0.9 cm^2^, used to calculate TEER (Ohms*cm^2^).

### 2.4 Fluorescence staining, imaging, and analysis

5-ethynyl-2′-deoxyuridine (EdU, 1 μg/mL) was added to the culture medium 24 h before sample fixation. Cells were fixed with Prefer fixative (Anatech Ltd., cat. No. NC9053360) for 20 min, permeabilized with 0.5% Triton X-100 at 20°C, and then blocked with 1% bovine serum albumin (BSA) for 1 h. Subsequently, integrated EdU was stained with sulfo-Cy5-azide (1.25 μg/mL) and then primary antibodies were added (Flomerfelt and Gress, 2016). In this study, a DNA stain (Hoechst 33,342, Sigma-Aldrich, cat. No. B2261) and antibodies for mucin-2 (MUC2, Santa Cruz, cat. No. sc-15334), cytokeratin-20 (KRT20, Cell Signaling Technology, cat. No. 13063S), epithelial cell adhesion molecule (EpCAM, Bioss USA, cat. No. bs-0593R), and vimentin (VIM, Santa Cruz, cat. No. sc-6260) were used (all 1:500 dilution). After overnight incubation at 4°C with primary antibodies, the samples were rinsed and matched secondary antibodies with Alexa Fluor 488 (goat anti-mouse, Life Technologies Corp., cat. No. A28175), and Alexa Fluor 555 (donkey anti-rabbit, Life Technologies Corp., cat. No. A31572) fluorophores were added and then rinsed away after overnight incubation at 4°C. Confocal microscopy was performed with an inverted Olympus Fluoview 3,000 equipped with 405, 488, 561, and 640 nm laser diodes and 4x, 10x, or 20x magnifying objectives were used to obtain images.

For image analysis in planar systems, Cell Profiler [cellprofiler.org (Stirling et al., 2021)] was used to segment and measure area occupied by each fluorescent marker. Planar samples were each imaged in three representative sections with the 20x objective and a maximum intensity projection of the z-stack (step size 3.93 µm) was analyzed. Three images from three technical replicates were each analyzed across three repeated experiments. For 3D crypts, 4x overview images were obtained from the top (z-stack, slice 25.4 µm) and a maximum intensity z-projection was used to analyze the number of crypts with EdU + cells. Next, the membrane was detached from the insert base and cut in half using microdissection scissors, the crypt area was situated perpendicular to the objective and imaged from the side. The 10x objective (step size 4 µm) was used to obtain images and the z-stacks were reconstructed in 3D using IMARIS X64 v9.8.2 (imaris.oxinst.com, Oxford Instruments) for analysis. The number and position of nuclei (Hoechst 33342+), and proliferative nuclei (EdU+) were counted using the IMARIS spots module, and the volume occupied by fibroblasts (vimentin+) was measured using the volume module.

### 2.5 Bulk RNA-sequencing

For gene expression comparison, RNA was extracted from 3D crypt arrays with monoculture epithelium, fibroblasts, or coculture epithelium with fibroblasts (n = 3). To capture only the tissue of interest within crypt arrays, a 3 mm biopsy punch was used to remove the *in vitro* crypt array within well-plate inserts. The extracted tissue portions were then agitated via vortexing and repeated pipetting within RNA lysis buffer (Zymo Research, cat. No. R1057) and then the RNA was extracted using a Quick-RNA™ MiniPrep Plus kit (Zymo Research, cat. No. R1057). Sequencing was then conducted on an Illumina NextSeq 2000 (Illumina. San Diego, CA). Software (STAR v2.7.7a), 2-pass mapping was used to align paired-end reads to human hg38 assembly and then GENCODE annotation v38 along with gene-level read quantification was performed (Dobin et al., 2013). Software (FastQC 0.11.9, RNA-SeQC 2.3.4, RSeQC 4.0.0) were used for quality control with assessment of insert fragment size, read quality, read duplication rates, rRNA rates, gene body coverage and read distribution in different genomic regions (Andrews, 2010; Deluca et al., 2012; Wang et al., 2012). Bioconductor package edgeR 3.36.0 was used to detect differential gene expression between sample groups (Robinson et al., 2010). Genes with low expression were excluded using the edgeR function filterByExpr with min. count = 10 and min. total.count = 15. The filtered expression matrix was normalized by trimmed mean of m-values (TMM) method and subject to significance testing using the quasi-likelihood pipeline implemented in edgeR. A gene was deemed differentially expressed if absolute log2 fold change was above 1 (*i.e.,* fold change >2 in either direction) and Benjamini–Hochberg adjusted *p*-values were less than 0.05.

### 2.6 Statistical analysis

For fluorescence and TEER data, a student’s t-test was conducted to assess the significant difference between epithelium-only and coculture conditions. Data are presented as mean ± standard deviation. Differences between means from separate groups (epithelium, fibroblasts and coculture) were determined using 2-way ANOVA multiple comparisons by Tukey’s multiple comparisons, unless otherwise specified. The level of significance is indicated as the *p*-value in each experiment. Asterisks in figures indicate: *, *p*-value < 0.05; **, *p*-value < 0.01; ***, *p*-value < 0.001, ****, *p*-value < 0.001; ns (not significant), *p*-value > 0.05. Statistical analysis and graphical illustrations were performed using GraphPad PRISM 9 software, version 9.5.0.

## 3 Results

### 3.1 Design of a platform for epithelial cell-fibroblast coculture

**FIGURE 1:**
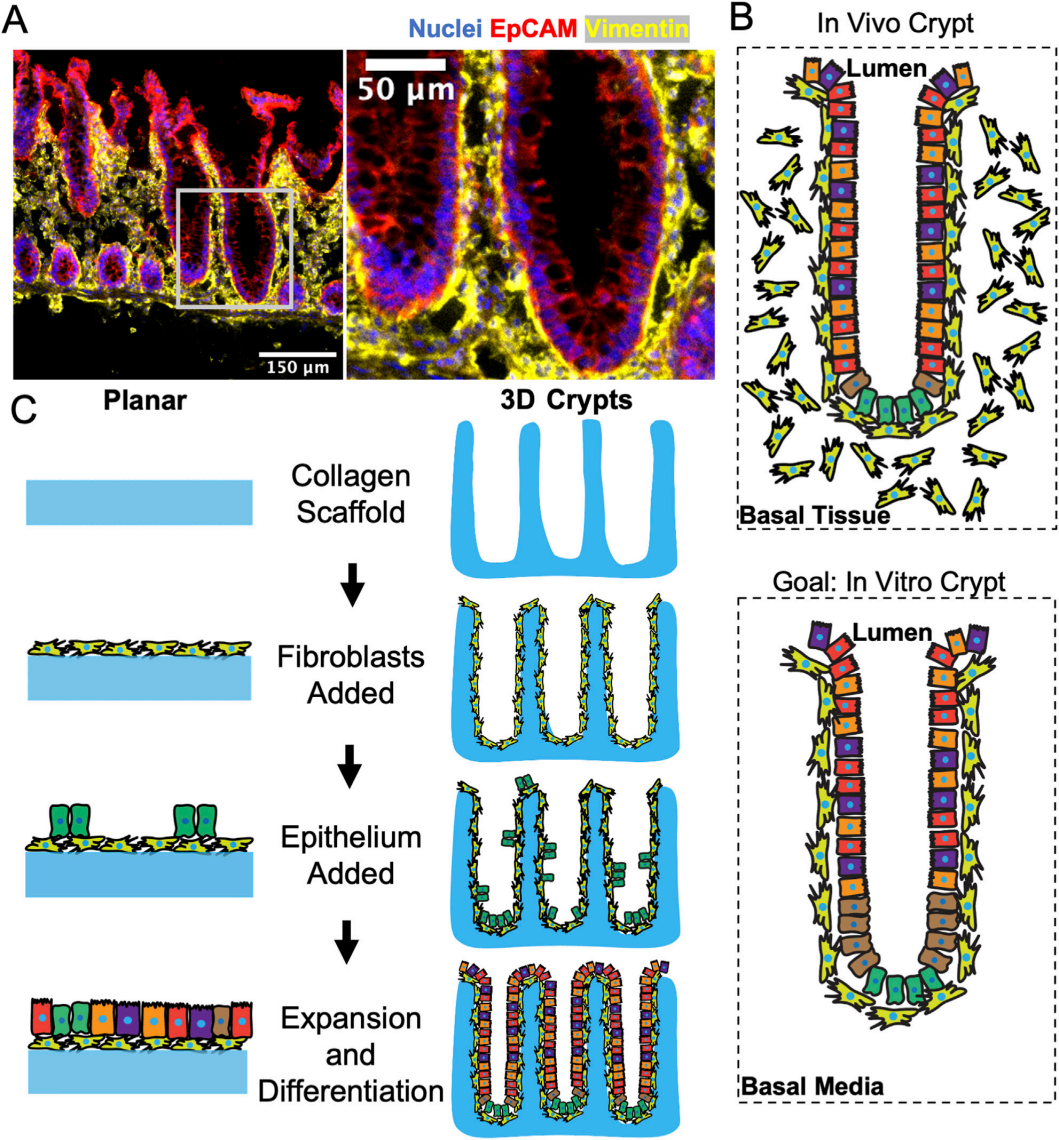
Model system to recapitulate the direct interactions of colonic fibroblasts with epithelium. **(A)** Fluorescence staining of a sliced section of human colon tissue displaying nuclei, epithelium, and fibroblasts. The white box in the left panel locates the higher magnification inset of the right panel. **(B)** Schematics showing the *in vivo* and *in vitro* positioning of columnar epithelial cells above pericryptal fibroblasts within a crypt. **(C)** Schematics of the sequential seeding process for generating a planar (2D) and crypt-shaped (3D) coculture model.

To visualize the *in vivo* location of the epithelial cells with respect to fibroblasts, human colonic tissue was fixed, sectioned, and immunostained for vimentin to identify fibroblasts and EpCAM to label epithelial cells. Fibroblasts were observed throughout the stroma beneath and between the colon crypts (Figures 1A, B). Importantly a layer of pericryptal fibroblasts was in close apposition to all epithelial cells including those within the crypts as well as those lining the luminal surface. To recapitulate this close relationship between the pericryptal fibroblasts and epithelial cells, two *in vitro* systems were developed (Figure 1C): *i)* a planar coculture model and *ii)* a 3D crypt coculture model. Both systems employed a scaffolding comprised of a cross-linked collagen that was molded into either a flat surface or a three-dimensional crypt array. The collagen scaffold in both flat and molded-crypt models was placed within a hanging basket to enable control of media conditions on both sides of the scaffolding (Wang et al., 2018a). The flat collagen slab supported planar growth of the two cell types and enabled rapid screening of coculture conditions to support both fibroblasts and epithelial cells. Once the optimal coculture conditions were determined, a shaped scaffolding to support the crypt arrays was constructed. Screens on the flat collagen slabs employed a tissue-cultured intestinal, fibroblast cell-line (CCD18-Co). The 3D crypt arrays used primary human colonic fibroblasts which are a more restricted resource, but which more closely resembled the *in vivo* cell type than the cell line.

### 3.2 Identification of common culture conditions for fibroblasts and epithelial cells

Fibroblasts and epithelial cells are typically cultured under distinct conditions. Fibroblasts grow well in commonly used media such as DMEM supplemented with serum while epithelial cells require a wide range of additives such as B27, Y-27632, A83-01, SB202190, and growth factors of Wnt, R-spondin, Noggin (WRN) and epithelial growth factor (EGF) to maintain stem cells and support their proliferation (Supplementary Table S1). Fibroblasts are typically cultured on a polystyrene surface [stiffness ∼3 GPa (Wypych, 1941)] while intestinal epithelial stem cells require a much softer matrix (e.g., collagen, Matrigel with stiffness of 0.1–1 kPa) with proper ECM contacts for cell survival (Wang et al., 2018b). An initial goal of this work was to identify a medium and surface on which both fibroblasts and epithelial cells would be viable and maintain their appropriate phenotype. Since fibroblasts are more tolerant of their culture conditions than epithelial cells, fibroblasts were initially cultured under conditions supportive of intestinal epithelial stem cells. Under these culture conditions, fibroblasts demonstrated poor survival and altered morphology (Supplementary Figure S1A). To understand which medium additive might be negatively impacting the cells, fibroblasts were cultured on polystyrene 12-well plates in fibroblast medium but with the addition of a single component from the epithelial medium. The impact of WRN, EGF, A83-01, and SB202190 was not assessed since epithelial stem/proliferative cells are critically dependent on these factors (Sato et al., 2011). Cell morphology and surface coverage were examined over time. Fibroblasts without the epithelial-cell additives spread across the culture surface and grew to confluence within 3 days while fibroblasts in the presence of B27 or Y-27632 possessed a spindle-shaped morphology and had either increased turnover or did not grow to cover the substrate (Supplementary Figure S1B). B27 is a serum-free cocktail of culture supplements originally tailored to optimize neuronal cell culture but commonly used in epithelial *in vitro* culture. Y-27632 is a Rho kinase inhibitor added to the epithelial media formulation to help epithelial cells survive while dissociated during thawing or passage (Han et al., 2017). Since B27 and Y-27632 altered fibroblast morphology and growth properties, both were removed from the medium. Primocin is a potent, broad-spectrum antibiotic used to eliminate fecal bacteria during initial colonic cell isolation and it was also replaced with more conventional tissue culture antibiotics, e.g., penicillin/streptomycin. All subsequent cultures for fibroblasts and/or epithelial cells used penicillin/streptomycin but not Primocin, B27, or Y-27632 (Supplementary Table S1).

**FIGURE 2:**
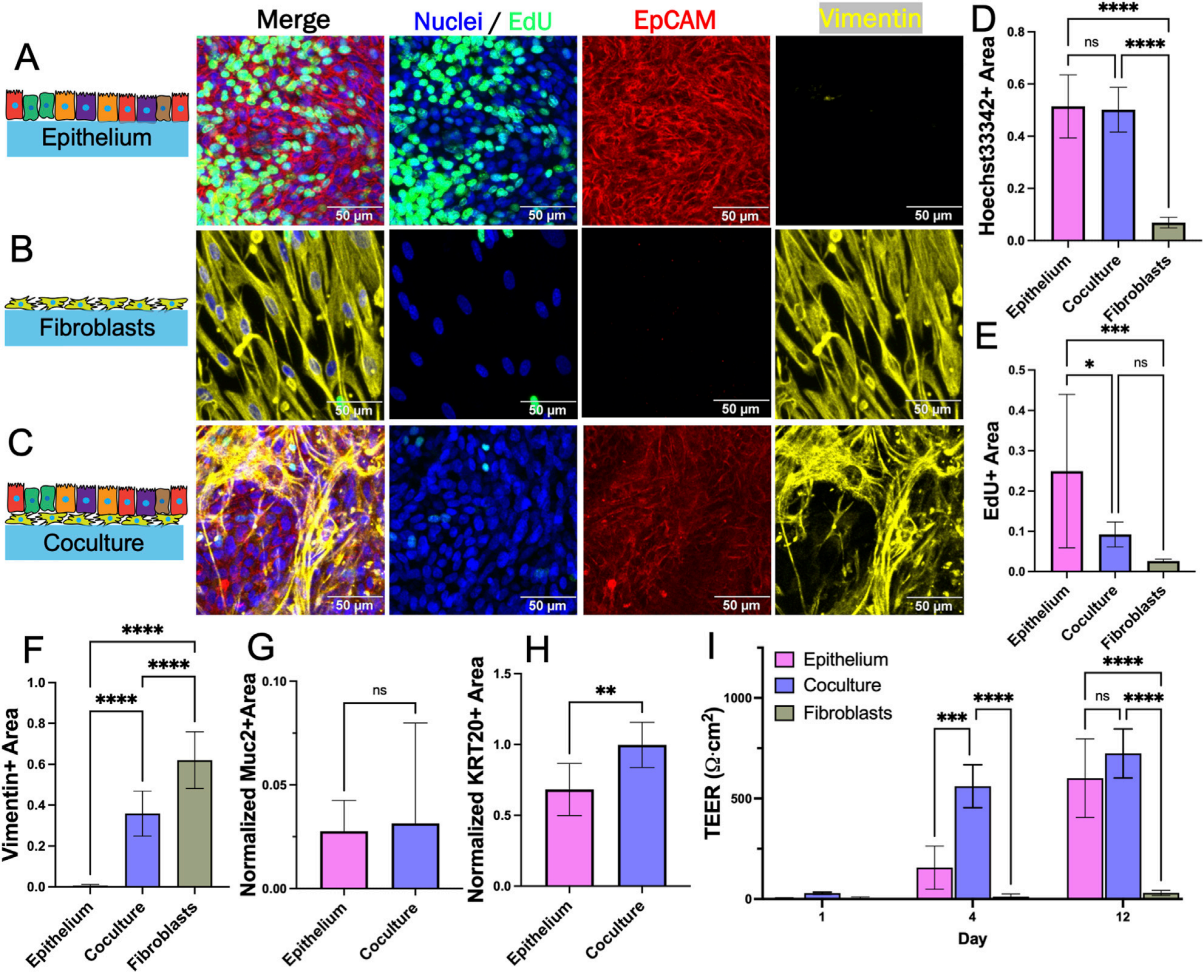
Evaluation of fibroblast and epithelial cell coculture on a 2D scaffold. **(A)** Epithelium alone after 8 days **(B)** fibroblasts alone after 7 days, and **(C)** coculture after 15 days total (7 days fibroblasts only, then 8 days after epithelium addition) in planar culture stained for Hoechst 33,342, EdU incorporation, and EpCAM and vimentin immunofluorescence. **(D–F)** The fluorescence area positive for **(D)** Hoechst 33,342 **(E)** EdU incorporation, and **(F)** vimentin staining was plotted for each culture system. The area positive for each of these markers was determined as that above an empirically set threshold. **(G–H)** Shown is the normalized fluorescence area positive for **(G)** mucin 2 and **(H)** KRT20 immunofluorescence. The normalized area was calculated by dividing the area positive for mucin 2 and KRT20 (above an empirically set threshold) with the area positive for Hoechst 33,342 fluorescence (above an empirically set threshold). **(I)** TEER (n = 4) measured on different days for the cultures. ns = not significant, * = *p*-value < 0.05, ** = *p*-value < 0.01, *** = *p*-value < 0.001, **** = *p*-value < 0.0001. Panels D–I display the average data with a single standard deviation marked by the error bar. In panels D–H, n = 3 biological replicates for each with 3 technical replicates for each sample.

To identify a common substrate on which both epithelial cells and fibroblasts could attach and grow, the two cell types were cultured separately, and together, on a flat cross-linked collagen surface (stiffness approximately 1 kPa) under the modified medium (Figures 2A–C). Cell density was assessed by measuring the area covered by nuclei (Hoechst 33342-stained DNA) and quantifying cell proliferation (EdU incorporation). Cell morphology was judged following immunostaining for EpCAM or vimentin. When cultured alone, epithelial cells grew to confluence with large numbers of proliferative cells (Figures 2D, E). Despite removal of Primocin, B27 and Y-27632, the epithelial cells grew well and displayed characteristic markers such as KRT20 and mucin-2 but not vimentin (Figures 2F–H). The fibroblasts also grew to cover the cross-linked collagen surface and very few proliferative cells were observed relative to that of epithelium alone (Figure 2E). Barrier function of the monocultures was assessed by measuring TEER over time. On day 1 after seeding, TEER was near zero and not significantly different between fibroblast and epithelium cultures (Figure 2I). On day 4 and 12, the TEER was significantly greater for the epithelial cells compared to the fibroblasts. The fibroblast and epithelial cells both displayed excellent surface coverage, expected functional behavior and acceptable morphology on the cross-linked collagen which was therefore used for all subsequent experiments.

### 3.3 Planar coculture of fibroblasts and epithelial cells in the presence of WRN

For coculture, fibroblasts were plated at day 0 on the cross-linked collagen surface and cultured in fibroblast medium for 7 days. Epithelial cells were then plated onto the fibroblast-covered collagen scaffold at day 7 and cultured in EM until the epithelial cells reached confluence above the fibroblasts (8 days) (Supplementary Figure S2A). The cocultures displayed both EpCAM+ and vimentin + cells indicating that both epithelial cells and fibroblasts were present on the scaffold (Figure 2C). The measured vimentin + area was significantly higher in coculture as compared to epithelium alone indicating that fibroblasts are present though there was a significant reduction in vimentin coverage for the coculture relative to that of the fibroblast only culture (Figure 2F). Fewer fibroblasts were present in the coculture relative to the fibroblast only culture despite plating identical numbers of cells on day 0 so the presence of the epithelial cells appeared to diminish the number of fibroblasts. This was surprising since these cells grow in proximity *in vivo*. The Hoechst 33342 + surface area was not statistically different for coculture as compared to epithelial cells alone (Figure 2D). The surface area occupied by EdU + cells was significantly lower for coculture as compared to epithelial cells alone (Figure 2E). The normalized expression of KRT20 (a pan-differentiation marker for epithelial cells) was significantly greater for cells in coculture compared to that of epithelial cells alone although Muc2 (a marker for goblet cells) was not significantly different (Figures 2G, H). The decreased EdU incorporation and increased KRT20 expression suggested that in this simple planar system, the fibroblasts act to decrease epithelial cell proliferation and encourage their differentiation. The TEER was significantly lower on day 4 for epithelial cells alone versus coculture (Figure 2I). A high TEER signifies the presence of mature colonocytes with tight cell-cell interconnections again suggesting that the fibroblasts promote epithelial cell differentiation towards colonocyte lineage. By culture day 12, TEER was not significantly different between monoculture and coculture suggesting that both systems eventually grew to confluence with a majority of colonocytes. These data suggest that the fibroblasts modulate epithelial cell proliferation while encouraging mature colonocyte differentiation and tissue barrier function (even in the presence of exogenous WRN). However, limitations of this simple planar coculture system include the absence of discrete cell zones and the high concentration of WRN throughout the culture so that subtle impacts of the fibroblasts on epithelial cells particularly the stem/proliferative cells might be obscured.

### 3.4 Planar coculture of epithelial cells with fibroblasts without exogenous WRN

**FIGURE 3:**
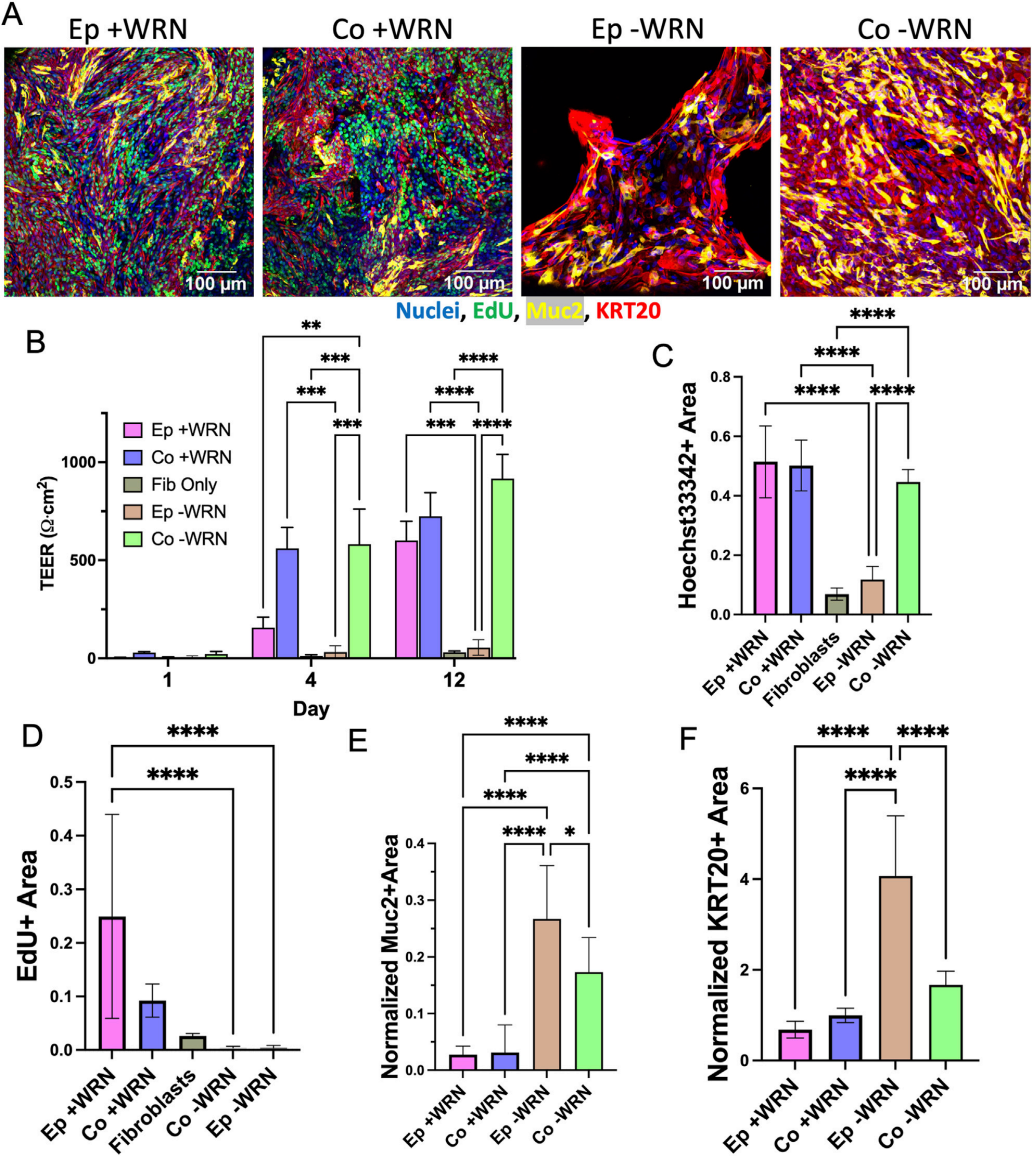
Impact of removal of exogenously added growth factors. **(A)** Epithelium with (Co.) and without (Ep) fibroblasts cultured in the presence or absence of growth factors (+/− WRN). Images taken 8 days after epithelium addition. **(B)** TEER measured over time for the epithelium monoculture and coculture after removal of exogenously added WRN. **(C–D)** The fluorescence area positive for **(C)** Hoechst 33,342, and **(D)** EdU incorporation was plotted for each culture method. The area positive for each of these markers was determined as that above an empirically set threshold. **(E–F)** The normalized fluorescence area positive for **(E)** mucin 2 and **(F)** KRT20 immunofluorescence. The normalized area was calculated by dividing the area positive for MUC2 and KRT20 (above an empirically set threshold) with the area positive for Hoechst 33,342 fluorescence (above an empirically set threshold). ns = not significant, * = *p*-value < 0.05, ** = *p*-value < 0.01, *** = *p*-value < 0.001, **** = *p*-value < 0.0001. Panels B–F display the average data with a single standard deviation marked by the error bar and n = 3 biological replicates with 3 technical replicates for panels C–F.

*In vivo*, a major source of Wnt supporting the colonic stem cell niche is pericryptal fibroblasts (Roulis and Flavell, 2016). To determine whether cocultured fibroblasts might provide supportive factors for the epithelial cells in this model, epithelial cells with or without fibroblasts were cultured in the absence of exogenous WRN. Just as before, fibroblasts were first cultured in simple fibroblast medium on the cross-linked collagen scaffold until confluent (5–7 days). Epithelium was then added above the fibroblasts for coculture or directly onto a collagen scaffold for monoculture and then cultured with optimized medium without WRN. A marked difference was observed in the cell coverage, proliferation, and lineage allocation of epithelium monoculture and coculture with and without WRN after 12 days in culture (Figure 3A). On the first day after addition of the epithelial cells, TEER was near-zero for all cultures (Figure 3B). On day 4 and 12 there was a significantly higher electrical resistance in the cocultures relative to that of day 1 as well as the epithelium only cultures on any day. Epithelial cells without WRN or fibroblasts were not able to proliferate to cover the scaffolding and thus never achieved an elevated TEER. In coculture, epithelial cells proliferated to cover the scaffold with an increased TEER, suggesting that fibroblasts were able to mitigate the absence of exogenous WRN to some degree. On day 12, Hoechst 33342-stained nuclei covered almost four times more area in the cocultured cells compared to epithelial cells alone, again indicating that the fibroblasts supported an increased epithelial cell number in the absence of WRN (Figure 3C). Notably, Hoechst 33342 coverage in coculture without WRN was not significantly different than previously measured coculture with WRN, indicating that these tissues reach similar levels of cell density. By day 12, both the coculture and epithelium monoculture possessed few EdU + cells, a significant decrease compared to both culture systems when exogenous WRN was provided (Figure 3D). These data suggest that the fibroblasts partially compensated for the absence of WRN. Next, the presence of differentiated cells on day 12 was examined by staining the cultures for KRT20 and Muc2. The normalized area occupied by both markers was significantly diminished in coculture as compared to monoculture (Figures 3E, F). The removal of exogenous WRN is known to induce rapid differentiation in epithelial cells and the presence of the fibroblasts partially mitigated this effect. Under these conditions fibroblasts appeared to support epithelial cells by slowing differentiation; however, pericryptal fibroblasts in this system did not mimic the full impact of exogenously added WRN. Thus, the fibroblasts appeared to be capable of supporting undifferentiated as well as differentiated epithelial cell states with the dominant effect dependent on the presence or absence of exogenous WRN. However, the absence of distinct cell compartments made these opposing impacts difficult to investigate motivating the construction of a 3D architecturally accurate model of the colonic crypt with an underlying layer of fibroblasts.

### 3.5 3D coculture of fibroblasts and epithelial cells in a colon crypt architecture

**FIGURE 4:**
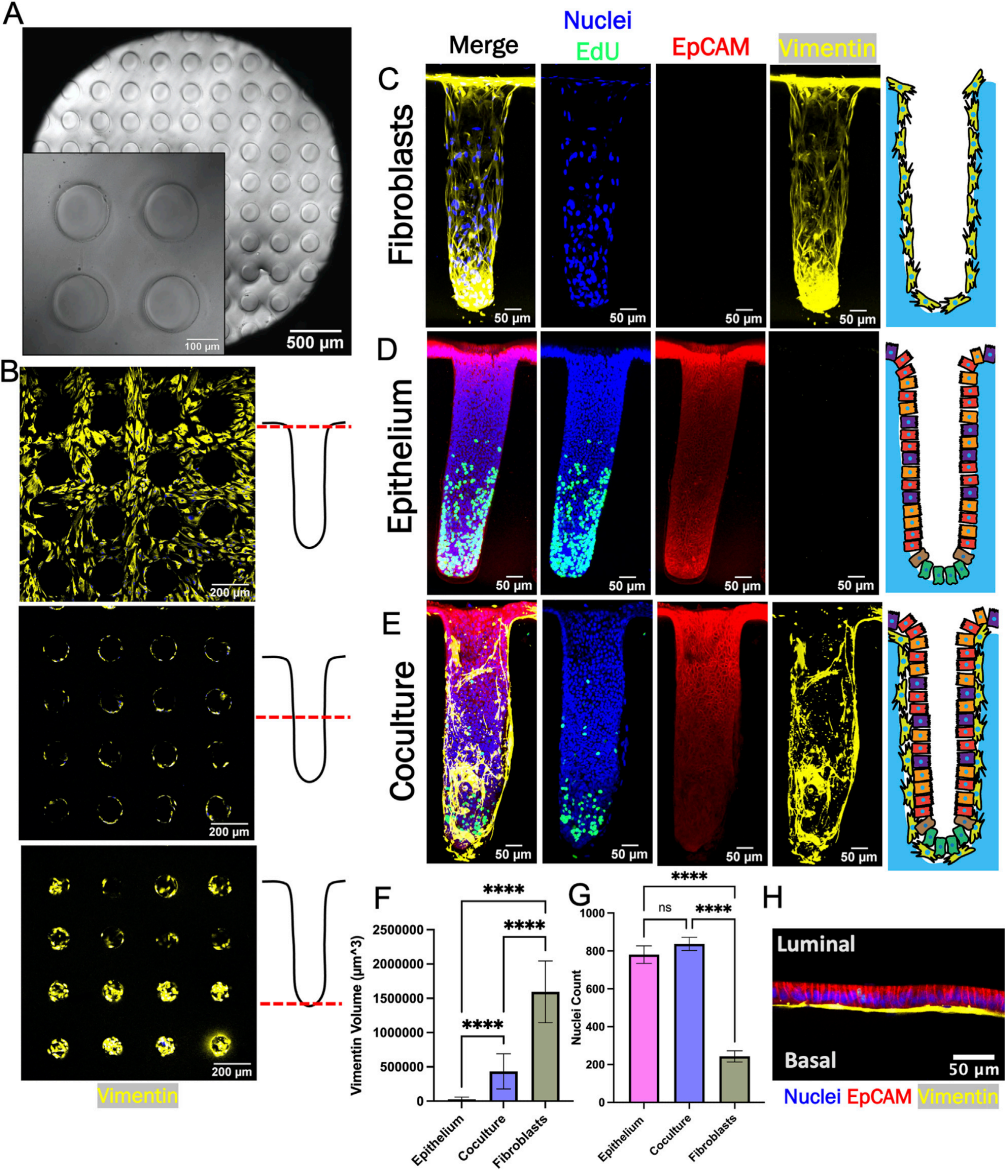
Epithelium and fibroblasts cultured as 3D crypts. **(A)** Brightfield top-view image of a fabricated collagen crypt array. **(B)** Fluorescence image of fibroblasts growing on a crypt array. Images were obtained at different focal planes (dashed red line) along the length of the crypt as shown in the accompanying schematics (right panels). **(C)** Sideview maximum z-projection of crypt with only fibroblasts. **(D)** Sideview maximum z-projection of a crypt with only epithelial cells. **(E)** Sideview maximum z-projection of a crypt with cocultured fibroblasts and epithelium. **(F)** Measured volumetric area (vimentin +) occupied by fibroblasts (n = 3 biological replicates, n = 34, 45, and 10 crypts for epithelium, coculture and fibroblast, respectively). The vimentin + area was that measured to above an empirically set threshold. **(G)** The total number of nuclei per crypts (n = 3 biological replicates, n = 34, 45, and 10 crypts for epithelium, coculture and fibroblast, respectively). **(H)** Confocal image (X-Z plane) of fibroblasts beneath epithelium (red = EpCAM immunofluorescence, blue = Hoechst 33,342, yellow = vimentin. Ns = not significant, **** = *p*-value < 0.0001. Panels F and G display the average data with a single standard deviation marked by the error bar.

To enable deeper insights into the fibroblast-epithelial cell interactions, crypt arrays were formed on a molded cross-linked collagen scaffold positioned within the luminal compartment of a hanging basket (Figure 4A; Supplementary Figure S3A). When fibroblasts were cultured on the arrays in fibroblast medium, the cells grew to confluence across the surface of the arrays and down into the microwells without occluding the microwells over 6–8 days (0% of crypt lumens obstructed, n = 88, Figure 4B, Supplementary Figure S2B, C). Fibroblasts expressed vimentin but not EpCAM and the cells established a continuous monolayer along the base and walls of crypts and over the inter-crypt luminal surface (Figures 4B, C). Additionally, little EdU incorporation was observed in fibroblast monoculture suggesting that the cells were very slow growing under these conditions.

For coculture, epithelial cells were placed atop fibroblasts after 7 days and then cultured for a further 12 days. Once the epithelial cells formed a confluent layer above the fibroblasts (day 8), the medium in the luminal reservoir of the hanging basket was replaced with differentiation medium without WRN (DM, Supplementary Table S1). Stem cell medium with WRN was maintained in the basal reservoir (SM, Supplementary Table S1). Prior work has demonstrated that a standing gradient of WRN is created along the long axis of the crypts under these conditions and that this gradient supports formation of a stem/proliferative cell niche and differentiated cell zone in epithelial cell only cultures (Figure 4D) (Wang et al., 2017a; Wang et al., 2018a; Villegas-Novoa et al., 2024). After culture for 4–5 days under the WRN gradient, cells on the coculture crypt arrays were assayed for proliferation and protein markers. As expected, EpCAM + cells were identified in coculture and epithelium-only cultures while vimentin + cells were present in coculture and fibroblast-only cultures (Figures 4C–E). The vimentin + cell volume in the crypt arrays was significantly lower for coculture crypts compared to fibroblast-only crypts suggesting that addition of the epithelial cells diminished the number of fibroblasts as observed on the planar scaffolds (Figure 4F). The number of nuclei (Hoechst 33342 + objects) was significantly greater for the epithelium only and coculture crypts relative to the fibroblasts as predicted by the planar culture results (Figure 4G). EdU+ cells were localized to the crypt base of the epithelium only and the coculture arrays suggesting that a WRN gradient successfully formed along the crypt long-axis under both conditions (Figures 4D, E). Finally, high magnification imaging revealed that flattened fibroblasts were established in close apposition to columnar epithelial cells within the 3D coculture (Figure 4H). Overall, the attributes measured for the crypt arrays are consistent with the trends observed in the planar model.

**FIGURE 5:**
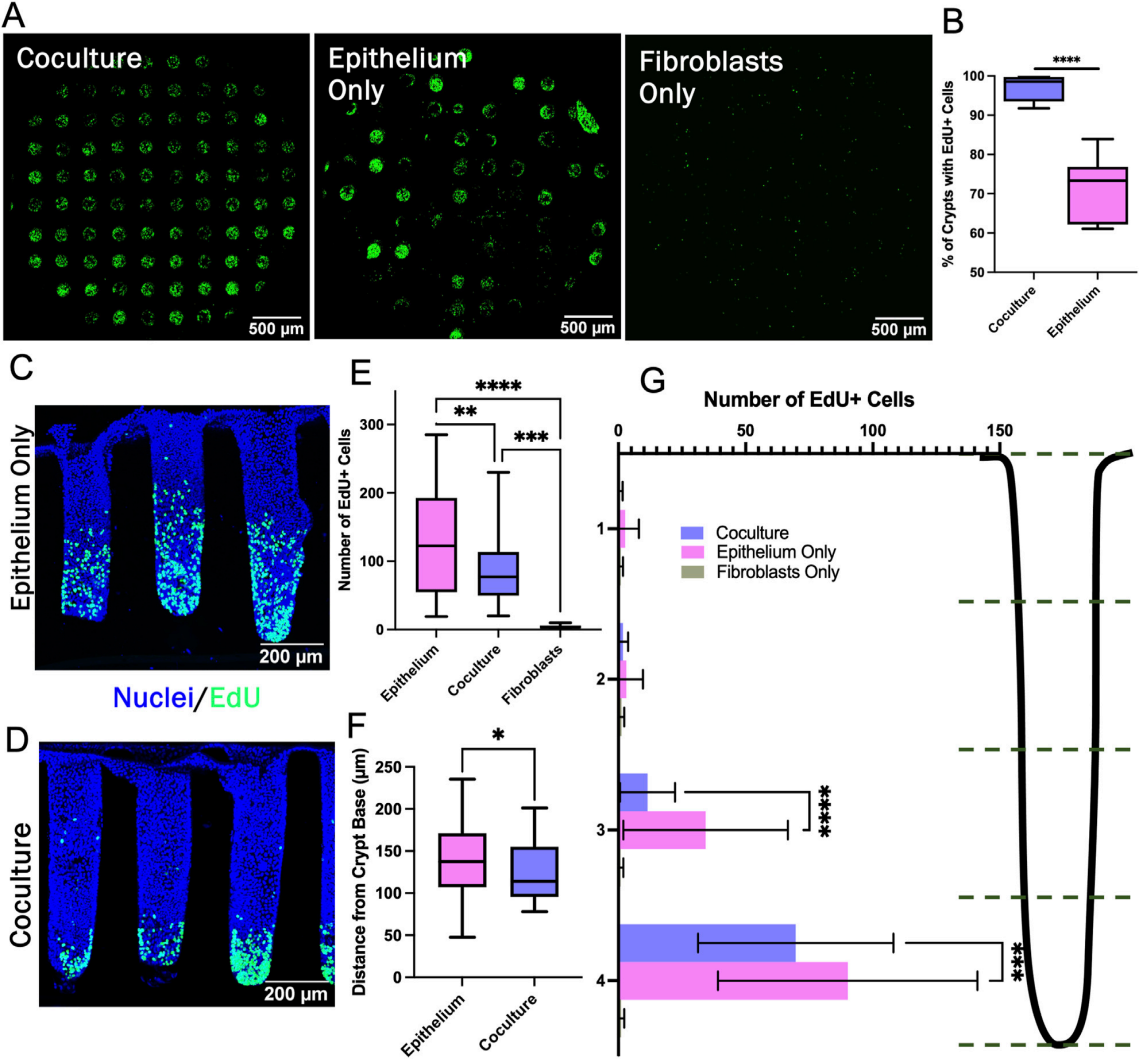
Characterization of the stem/proliferative cell zone. **(A)** Confocal image stack maximum z-projection of full crypt-arrays displaying cells that have incorporated EdU (green). **(B)** The percentage of crypts containing at least one visible EdU + cell (n = 5 biological replicates, i.e., full crypt arrays, n = 443 individual coculture crypts, n = 410 epithelium monoculture crypts). **(C–D)** Sideview of a representative epithelium only and coculture crypt stained with Hoechst 33,342 and for EdU incorporation. **(E)** The number of EdU + cells/crypt (n = 3 biological replicates, n = 34, 45, and 10 individual crypts for epithelium, coculture and fibroblast, respectively). **(F)** The average position of EdU + cells within individual crypts (n = 3 biological replicates, n = 34 individual crypts for epithelium and 45 for coculture). **(G)** The number of EdU + cells/crypt location plotted for four regions along the vertical axis of the crypts. Bins correspond to the accompanying schematic, i.e., bin 4 represents the bottom quartile of a crypt. * = *p*-value < 0.05, ** = *p*-value < 0.01, *** = *p*-value < 0.001, **** = *p*-value < 0.0001. Box plots in panels B, E, and F display the mean value marked by the central horizontal bar while the box displays the extents of the middle two quartiles (25%–75%) and the whiskers mark the minimum and maximum. Panel G displays the average data with a single standard deviation marked by the error bar.

To understand the impact of the fibroblasts on the stem/proliferative cell zone, 89 individual crypts across 27 arrays were characterized with respect to the number and location of the EdU + cells. The number of crypts in the coculture arrays possessing at least one EdU + cell was significantly greater than that of arrays with only epithelial cells (Figures 5A, B). Since the arrays were seeded with similar numbers of cells, the large number of epithelium-only crypts without an EdU + cell was most likely due to a loss of cells competent to divide. In contrast, the underlying fibroblasts in coculture arrays appeared to help epithelium to maintain a more durable stem/proliferative cell compartment on the arrays. For crypts that did possess at least one EdU + cell, the number of EdU + cells/crypt was highly variable for both culture systems and crypt-to-crypt variation was observed within all samples (Figures 5C, D). Crypts with fibroblasts supporting the epithelial cells possessed a range of 20–230 EdU + cells/crypt while the epithelial cell-only cultures possessed a range of 19–285 EdU + cells/crypt. However, the mean number of EdU + cells/crypt in the epithelial cell-only crypts was significantly greater than that of the coculture crypts (Figure 5E). Interestingly, although more of the epithelium-only crypts had zero EdU + cells, those crypts with at least one EdU + cell possessed a greater number of proliferative cells compared to crypts with underlying fibroblasts. Thus, the fibroblasts appear to act as a stabilizing influence on the proliferative cells supporting their survival yet diminishing their proliferation.

The impact of the fibroblasts on the spatial extent of the stem/proliferative cell zone was assessed for crypts that possessed at least one EdU + cell. The distance between each EdU + nucleus and the crypt base was measured. Significantly more EdU + cells were found within the bottom half of epithelium-only crypts compared to the fibroblast supported crypts, and the average location of the EdU + nuclei in the fibroblast-supported crypts was significantly closer to the crypt base than for crypts with only epithelial cells (Figures 5F, G). In addition to modulating the numbers of EdU + cells, the fibroblasts diminished the height of the proliferative cell zone along the crypt long axis creating a more compact proliferative cell zone. The 3D crypts revealed the subtle differences in the impact of fibroblasts on the proliferative cell compartment not observable in the simple planar model.

### 3.6 Bulk RNA-sequencing reveals differential gene expression in coculture

**FIGURE 6:**
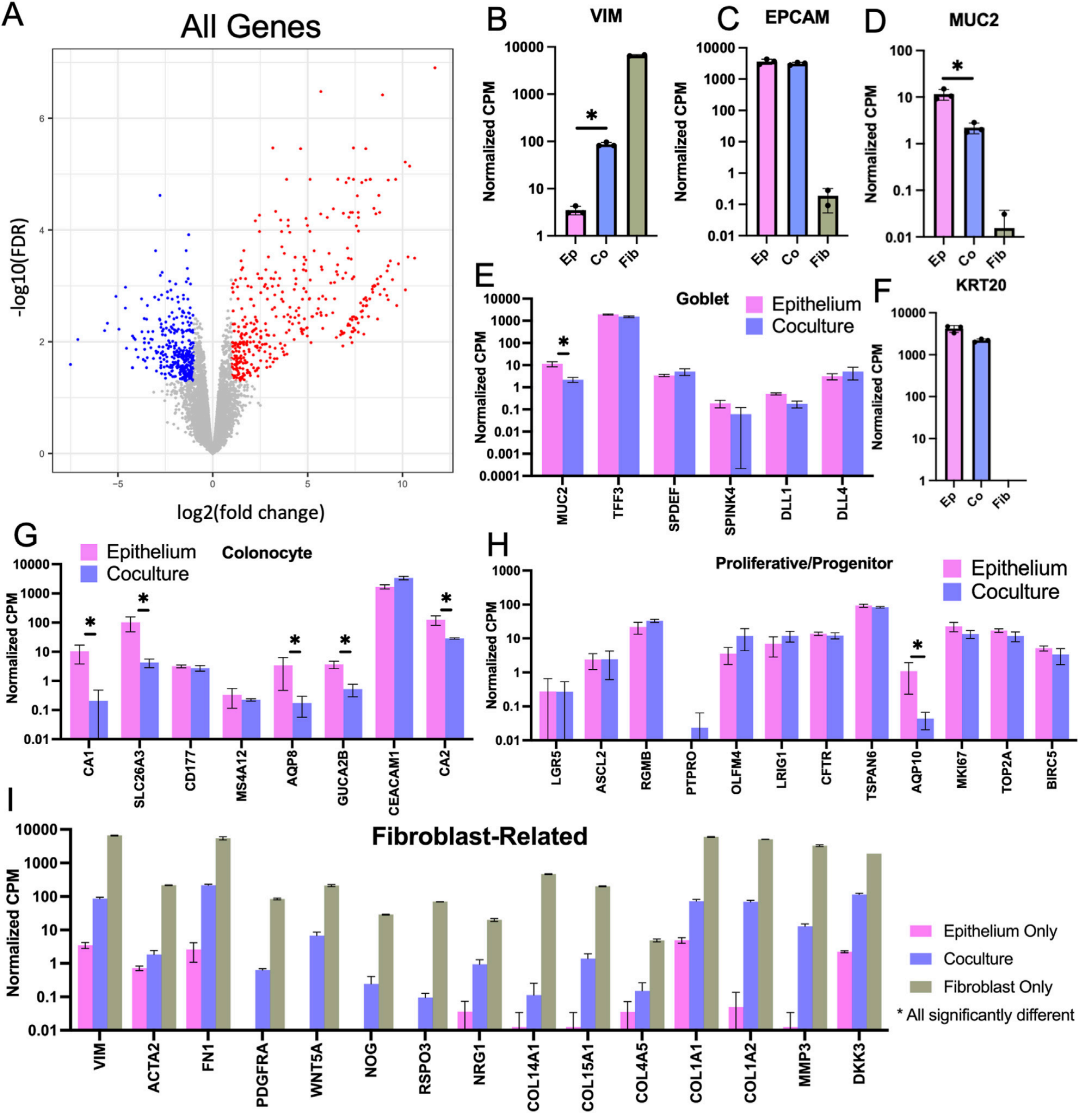
Bulk RNA-sequencing cells from 3D crypt arrays. **(A)** Volcano plot comparing epithelium versus coculture that displays the fold change (FC) and false discovery rate (FDR) for all 12,844 genes measured with red indicating significantly upregulated genes in coculture and blue signaling significantly downregulated genes in coculture. **(B–D)** Normalized counts per million (CPM) for mRNA expression of cell-type specific markers was compared for cultures of fibroblasts only, epithelial cells only and fibroblast-epithelial cell coculture. **(B)** Vimentin (fibroblasts). **(C)** EpCAM (all epithelial cells). **(D)** Muc2 (goblet cells). **(E)** Expression levels of additional goblet cell-specific mRNA transcripts. **(F)** Cytokeratin 20 (KRT20, differentiated epithelial cells). Expression levels of genes marking **(G)** colonocytes (differentiated absorptive epithelial cells) **(H)** progenitor cells (proliferative and not fully differentiated epithelial cells, including transit amplifying cells), and **(I)** Fibroblast-related genes, statistically significant difference detected in all displayed genes for in panel I (Brügger and Basler, 2023). N.b., * denotes statistical significance between epithelium monoculture and coculture only, as determined by Benjamini–Hochberg adjusted *p*-values from comparison of expression fold change.

To understand how the fibroblasts support the stem/proliferative cell compartment yet enhance differentiation, bulk RNA was isolated from whole crypt arrays with epithelium alone or fibroblasts and epithelium in coculture. mRNA for 12,844 protein-coding sequences was detected and compared (Figure 6A). Vimentin (*VIM*) mRNA, a fibroblast marker, was present only in cultures with fibroblasts while *EPCAM* mRNA encoding epithelial cell adhesion marker, a pan-epithelial cell marker, was expressed only when epithelium was present (Figures 6B, C). Of the 12,844 genes detected, 305 genes were significantly downregulated, and 379 genes were significantly upregulated in coculture when compared to epithelial monoculture (Supplementary Tables S2, S3). To identify which of these differentially expressed genes might be most informative, genes expressed predominantly in differentiated epithelial cells were first examined. *MUC2* mRNA encoding mucin-2 (MUC2), a major constituent of colonic mucus produced by goblet cells was highly expressed in both the coculture and epithelium only samples but with significantly greater *MUC2* expression in the epithelium only culture relative to that of the coculture sample (Figure 6D). Other goblet cell markers such as *TFF3* (trefoil factor 3), *SPDEF* (SAM pointed domain containing ETS transcription factor), *SPINK4* (serine peptidase inhibitor Kazal type 4), *DLL1* (delta like canonical Notch ligand 1), and *DLL4* (delta like canonical Notch ligand 4) were also expressed in epithelial cell cultures with and without fibroblasts but without a significant difference suggesting that goblet cell numbers may not have been altered in the presence of the fibroblasts (Figure 6E) (Dalerba et al., 2011). *KRT20* mRNA encoding for cytokeratin-20, a marker for all differentiated colonic epithelial cells, was abundantly expressed in both coculture and epithelium samples with no significant difference (Figure 6F). mRNA markers of colonocytes (absorptive cells) such as *CA1* (carbonic anhydrase 1), *CA2* (carbonic anhydrase 2), *SLC26A3* (solute carrier family 26 member 3), *AQP8* (Aquaporin 8), and *GUCA2B* (guanylate cyclase activator 2B) were downregulated in coculture relative to that of the epithelium alone suggesting that the cocultures might possess fewer mature colonocytes in the presence of the fibroblasts (Figure 6G) (Dalerba et al., 2011).

Next, markers of stem/progenitor intestinal cells were examined. *LGR5* (leucine rich repeat containing G protein-coupled receptor 5) mRNA, a marker for colonic stem cells, was present at low counts and without significant difference for epithelial cell cultures with and without fibroblasts. *OLFM4* (olfactomedin 4) mRNA, a marker for colonic transit amplifying cells in humans (Breau et al., 2022), was also present at low counts and without significant difference for epithelial monoculture and coculture. Of the 12 progenitor genes examined, only *AQP10* (Aquaporin 10) mRNA, a marker for colonic progenitor cells was significantly different (downregulated) in coculture relative to epithelium alone (Figure 6H) (Dalerba et al., 2011). Due to the very low numbers of stem/proliferative cells in all the cultures, differences in the stem/proliferative cell gene expression with and without fibroblasts may have been masked by the very large numbers of differentiated epithelial cells in the cultures. Single-cell mRNA expression analysis may be required to see these low frequency stem/progenitor cell types.

To examine a broader set of epithelial genes and remove confounding impacts of the fibroblast gene expression, all genes highly expressed in fibroblast monoculture (>1.0 CPM) were removed from the data sets obtained from epithelial cells with or without fibroblasts. Of the original 12,844 genes, 1835 filtered genes remained with 190 significantly downregulated and 43 significantly upregulated in coculture compared to the epithelial monoculture (Supplementary Figure S4A; Supplementary Tables S4, S5). A GO Analysis (geneontology.org) was conducted with the 43 upregulated genes to determine whether common biological processes might be identified, and genes related to negative regulation of endopeptidase activity were overrepresented in the gene set upregulated in coculture compared to that epithelium monoculture (Supplementary Table S6). Endopeptidase activity is associated with rapid proliferation with a need to recycle cellular constituents and this aligns with the observed increased proliferation in epithelium-only crypts where a stem/proliferative zone is maintained. A GO Analysis was also conducted for the 190 downregulated genes in coculture compared to epithelium monoculture and several transport related gene groups were overrepresented, i.e., triglyceride transport, water transport, and long-chain fatty acid transport (Supplementary Table S7). These transport genes are associated with a mature differentiated colonocyte consistent with the increased differentiation of the epithelial cells in the presence of fibroblasts observed in planar culture (Larrinaga et al., 2014).

Next, genes commonly expressed by fibroblasts with the potential to modulate epithelial cell behavior were examined in epithelium alone, fibroblasts alone, and coculture conditions (Brügger and Basler, 2023). As expected, transcripts for hallmark fibroblast markers such as *VIM* and α-smooth muscle actin 2 (*ACTA2*) and platelet derived growth factor-α (*PDGRFA*) were highly expressed by fibroblasts alone and in coculture (Figure 6I). The fibroblasts were also actively modifying their microenvironment as evidenced by mRNA expression of fibronectin, collagens, and matrix metalloprotease (*FN1, COL15A1, COL14A1, COL4A5, COL1A1, COL1A2, MMP3*. Figure 6I). Epithelial cell physiology is known to be regulated by ECM with components such as collagen and fibronectin regulating adhesion, morphology and proliferation (Onfroy-Roy et al., 2020). A variety of transcripts encoding growth factors were found to be expressed in fibroblast monoculture as well as the epithelium coculture. R-spondin 3 (*RSPO3*) participates in stem cell maintenance and drives proliferation while Noggin (*NOG*), a bone morphogenetic protein (BMP) antagonist, inhibits differentiation (Brügger and Basler, 2023). Wnt5a was highly expressed by fibroblasts alone and in coculture. This Wnt ligand associated with non-canonical Wnt signaling is often found to be expressed by luminal fibroblasts. It has a context-dependent effect though in the upper crypt region it decreases beta-catenin signaling thus encouraging epithelial differentiation rather than proliferation (Mikels and Nusse, 2006; Miyoshi et al., 2012; Miyoshi, 2017; Chalkidi et al., 2022; Brügger and Basler, 2023). Other transcripts identified include Neuregulin 1 (*NRG1*) which drives secretory cell formation and dickkopf 3 (*DKK3*), a WNT signaling pathway inhibitor. Taken together the presence of these transcripts indicate that the fibroblasts actively alter the microenvironment of the adjacent epithelial cells to modulate epithelial cell physiology.

## 4 Discussion and conclusion

In the large intestine, fibroblasts are found in close association with the epithelial cells throughout the crypt as well as in the luminal, inter-crypt space (Roulis and Flavell, 2016). To mimic this close *in vivo* colonic fibroblast-epithelial cell interaction, primary intestinal fibroblasts were cultured as a supporting layer of cells just below and in contact with primary intestinal epithelial cells. Two models with the 2 cell types in apposition were employed; an easy-to-build and assay monolayer as well as a more complex 3D model with the ability to reveal greater insights into the inter-cell type connectivity. The two systems were complementary with the monolayer enabling fast screening of culture conditions with facile assay of outcomes while the 3D crypt array was lower in throughput but provided much richer information. In both systems, enhanced epithelial differentiation was observed, and the overall rate of epithelial cell proliferation was diminished while in coculture with fibroblasts. Fibroblasts, on the other hand, exhibited reduced coverage when in coculture with epithelium, perhaps a result of increased competition with metabolically active epithelial cells for oxygen, nutrients, or adhesion sites. In the absence of exogenously provided growth factors, fibroblasts appeared to enhance epithelial cell survival, though the presence of pericryptal fibroblasts alone did not provide sufficient support for sustained epithelial cell proliferation, i.e., replace the added growth factors. Consistent with this effect was that the presence of fibroblasts appeared to support the maintenance of proliferative cells at the base of the crypts as evidenced by the presence of EdU + cells in nearly every crypt across an array of 3D crypts. Notably, this system does not incorporate fibroblasts throughout the lamina propria as observed *in vitro*, and so the ratio of fibroblasts to epithelial cells in the microenvironment is very low compared to the *in vivo* ratio. Additionally, these models do not attempt to incorporate numerous other cell types which impact the colon crypt niche like endothelial, immune, and neural cells nor does the system address the role of the gut microbiome. That said, in the microenvironment described herein, pericryptal fibroblasts diminished proliferation rate while promoting epithelial cell survival as well as enhancing the physiologic function of the differentiated cells, i.e., increased TEER.

Bulk RNA-sequencing of full crypt arrays revealed differential mRNA expression in coculture, compared to that of epithelial cells cultured without fibroblasts. Genes that were differentially expressed in coculture when compared to epithelium monoculture revealed potential mechanisms for fibroblast influence (Brugger et al., 2020). Secreted frizzled related protein 1 (*SFRP1*), a ligand which modulates canonical Wnt-signaling to diminish proliferation of intestinal epithelial cells, was upregulated in coculture relative to that in epithelial cell monoculture (Nik et al., 2013; Roulis and Flavell, 2016). Conversely, the mRNA of five solute carriers (*SLC6A19, SLC30A10, SLC17A4, SLC3A1, SLC36A1*) and three ATP-binding cassette transporters (*ABCG2, ABCB1, ABCG1*) was significantly downregulated in coculture relative to that in the epithelial cell monoculture. These transporter proteins, typically found on epithelial cells throughout the digestive tract, are responsible for translocation across membranes of specific molecules (i.e., amino acids, phosphate, manganese, *etc.*) or non-specific foreign substrates (Xie et al., 2018). This suggests that the fibroblasts can modulate intestinal absorptive behavior. Additionally, two genes which are related to apoptosis (*DAPK2, PRAP1*) were significantly downregulated in coculture relative to the epithelial monoculture. Death-associated protein kinase 2 (*DAPK2*), normally found in the intestinal epithelium, stimulates programed cell death while proline-rich acidic protein 1 (*PRAP1*), also expressed by intestinal epithelium, helps protect cells from oxidative stress-induced apoptosis (Wolfarth et al., 2020; Chen and MacDonald, 2023). While these downregulated genes may have opposing influences, the altered expression levels indicate that programmed cell death is modulated by fibroblasts. Bulk RNA-sequencing of these *in vitro* tissue constructs provides an overview of the altered microenvironment, though the interpretation of these results is limited by the bulk format. For coculture samples, the 2 cell types (epithelial and fibroblasts) are intermixed and a complex interplay exists with each cell type modulating the other’s gene expression. We provide sequencing data and have worked to glean information from genes specific to epithelial cells alone since we believe these insights are valuable for future work. With precise control of culture conditions and highly repeatable scaffold construction capabilities, the system will enable future experiments focused on single-cell mRNA expression to fully dissect the interplay of these cell types.

In addition to directing growth and differentiation, fibroblasts play key roles throughout the body by synthesizing and secreting ECM to provide a growth surface and support structure for other cell types. Intestinal fibroblasts are known to secrete collagen, laminin, and fibronectin as well as matrix metalloproteinases and they exert physical contractile forces all to build-up or reshape the basement membrane and interstitial space in health and disease (Chalkidi et al., 2022; Brügger and Basler, 2023). Within this model, fibroblasts were actively synthesizing ECM as suggested by the expression of ECM-related genes (e.g., *FN1, COL12A1, FBN1, FMOD*) in both the coculture and fibroblast monoculture. Matrix metalloproteinase 3 (*MMP3*), a protein that degrades collagen, fibronectins and laminins, is expressed by fibroblasts during development and remodeling within the colon and was highly expressed in coculture and fibroblast monoculture (Chalkidi et al., 2022). In the colon crypt *in vivo*, a gradient in fibroblast physiology is thought to exist along the long axis of the crypt with fibroblasts in different crypt regions producing unique ECMs as well as growth and differentiation factors (Brugger et al., 2020). These distinct fibroblast behaviors support the epithelial cells as they proliferate in the crypt base and then migrate towards the luminal surface developing into mature differentiated epithelial cells. The developed models did not investigate this gradient of fibroblasts but rather placed fibroblasts randomly across the scaffolding surface. With further development of this model, it should be possible to tailor fibroblasts behavior along the crypt long axis to display a gradient of phenotypes as occurs *in vivo*.

This *in vitro* microphysiological system with a 3D polarized colonic crypt represents a step forward towards a better understanding of the interplay between intestinal fibroblasts and epithelium within an *in vivo* like architecture. In the living human, many cell types are in proximity to the crypt stem cell niche making studies of the interactions between fibroblasts and the proliferative epithelial cells challenging, though evidence suggests that this relationship is paramount to intestinal health. The current system represents an improvement over prior *in vitro* models since primary intestinal-derived fibroblasts and epithelial cells are placed in direct apposition on the curved surface of and confined space of a crypt. This unique tool mimicking key colonic architectural features will enable future *in vitro* modelling of diseases in which epithelial cells and fibroblasts conspire to create a pathological outcome.

## Data Availability

The datasets presented in this study can be found in online repositories. The names of the repository/repositories and accession number(s) can be found in the article/Supplementary Material.
