# Supplementary material for: Fibroblasts modulate epithelial cell behavior within the proliferative niche and differentiated cell zone within a human colonic crypt model
Source: Front Bioeng Biotechnol. 2024 Dec 16;12:1506976. doi: 10.3389/fbioe.2024.1506976 (PMC11683563; doi:10.3389/fbioe.2024.1506976)
Supplement: Supplementary file 1 [file DataSheet1.docx]

***Supplementary Material***

**Fibroblasts modulate epithelial cell behavior within the proliferative niche and differentiated cell zone within a human colonic crypt model**

Angelo Massaro ^a^, Cecilia Villegas Novoa ^a^, Yuli Wang ^a^, Nancy L. Allbritton ^a,^ *

^a^ Department of Bioengineering, University of Washington, Seattle, WA 98195, USA

*Corresponding Author: [nlallbr@uw.edu](mailto:nlallbr@uw.edu)

**Supplementary Material**

Table S1………………………………………………………………………. Media composition

Table S2 (.xls file)………………………………………………...List of all downregulated genes

Table S3 (.xls file)……………………………………………………List of all upregulated genes

Table S4 (.xls file)……………………………………………List of filtered downregulated genes

Table S5 (.xls file)………………………………………………List of filtered upregulated genes

Table S6 (.xls file)…………………………...GO Analysis of filtered downregulated genes (190)

Table S7 (.xls file)………………………………..GO Analysis of filtered upregulated genes (43)

Figure S1……………………………………………… Fibroblast tolerance to media components

Figure S2………………………………………………………Timeline for *in vitro* tissue seeding

Figure S3……………………………...Well-insert fabrication & Fibroblast addition to 3D crypts

Figure S4…………………………………….Volcano plot of filtered differential gene expression

| Component (volume for 500 mL) | MM | EM | SM | DM |
| --- | --- | --- | --- | --- |
| L-WRN conditioned medium | 250 mL | 250 mL | 250 mL |  |
| Advanced DMEM/F12 (1x, Thermo Fisher Scientific, cat. no. 12634010) | 250 mL | 250 mL | 250 mL | 500 mL |
| Glutamax (100x, Thermo Fisher Scientific, cat. no. 35050061) | 5 mL | 5 mL | 5 mL | 5 mL |
| HEPES (1M, Corning, cat. no. 25-060-CI) | 5 mL | 5 mL | 5 mL | 5 mL |
| Primocin (50 mg/ml, InvivoGen, cat. no. ant-pm-2) | 500 µL | (5 mL Pen/Strep) | (5 mL Pen/Strep) | (5 mL Pen/Strep) |
| N-Acetyl-L-cysteine (NAC, 1M, MP Biomedicals, cat. no. 19460) | 500 µL | 500 µL | 500 µL | 500 µL |
| Epithelial growth factor (EGF, 250 µg/ml, PeproTech, cat. no. 315-09) | 100 µL | 100 µL | 100 µL | 100 µL |
| Nicotinamide (1M, Millipore-Sigma, cat. no. N0636) |  | 5 mL |  |  |
| B27 (50x, Thermo Fisher Scientific, cat. no. 17504044) | 10 mL |  |  |  |
| Gastrin (1 mg/ml, AnaSpec, cat. no. AS-64149) | 12.5 µL | 12.5 µL |  |  |
| Prostaglandin E_2_ (PGE2, 1 mM, Cayman Chemical, cat. no. 14010) |  | 5 µL |  |  |
| A 83-01 (5 mM, Millipore-Sigma, cat. no. SML0788) | 50 µL |  | 50 µL | 50 µL |
| SB202190 (30 mM, LC Laboratories, cat. no. S-1700) | 50 µL | 50 µL |  |  |
| Y-27632 (10 mM, MedChemExpress, cat. no. HY-10583) | 500 µL |  |  |  |
| Fetal bovine serum (FBS, 10%, R&D Systems, cat. no. S11150) |  |  |  | 50 mL |

Fibroblast Medium (FM) consists of 500 mL DMEM, 50 mL FBS, and 5 ml Penn/Strep

**Supplemental Table S1.** Media composition. MM: Maintenance medium. EM: Expansion medium. SM: Stem medium. DM: Differentiation medium. Adapted from Hinman et al. (Hinman *et al.*, 2021).


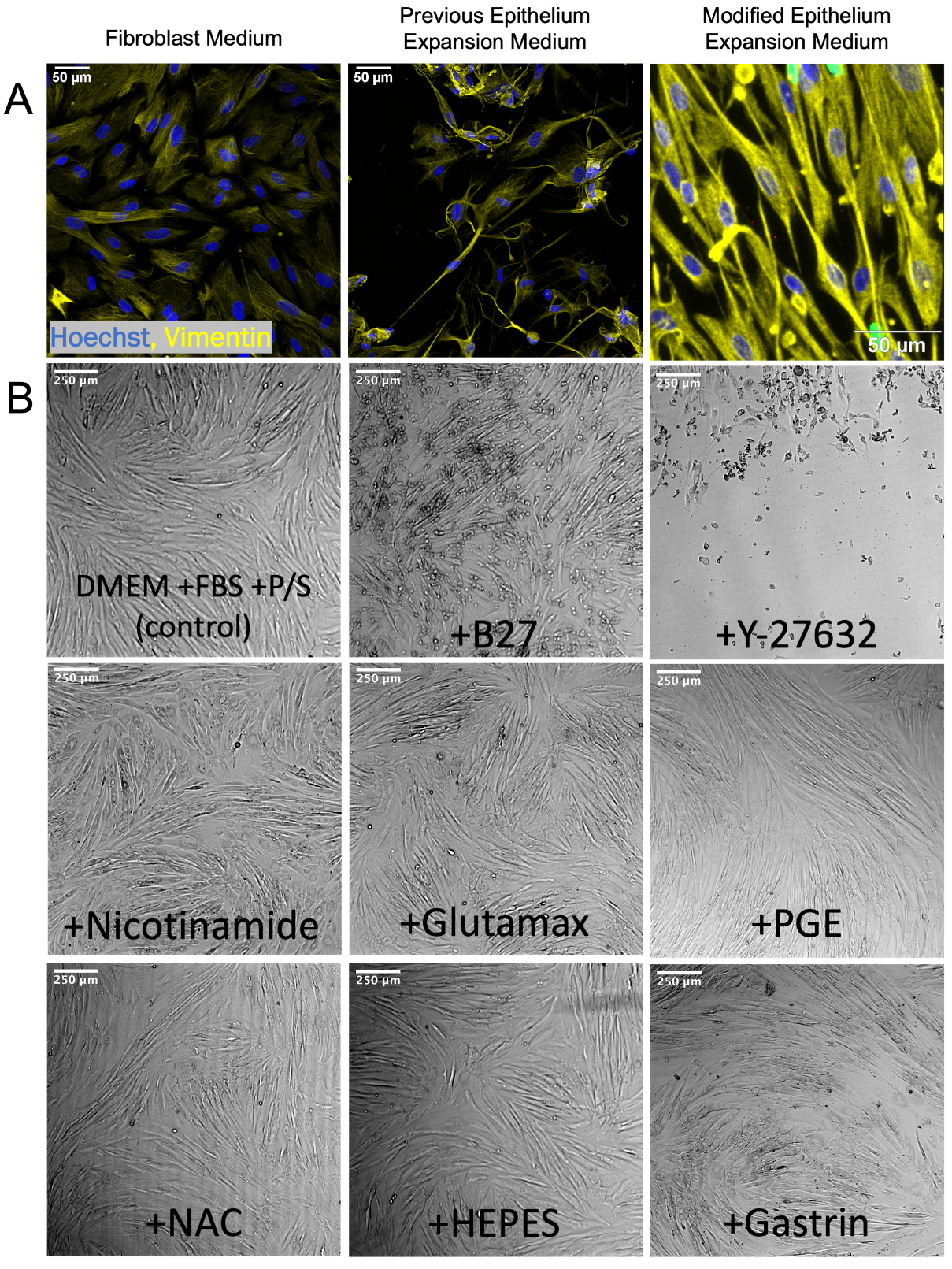


**Supplemental Figure S1.** Testing media components’ impact on fibroblasts. (A) Fluorescence images of fibroblasts stained for Vimentin and Hoechst 33342 to display cell shape and nuclei, respectively. All samples are grown on polystyrene within a 12-well plate with the left top panel displaying fibroblasts grown in the recommended monoculture media (DMEM with 10% FBS and 1% Penn/Strep. In the center top panel fibroblasts were grown with epithelial expansion medium optimized for epithelial monoculture and cell density is diminished while cell shape is altered. In the right-top panel fibroblasts were grown in modified expansion medium (EM) as indicated in Table S1. (B) Brightfield images of fibroblasts grown on polystyrene in fibroblasts medium (FM, control) plus one additional media component, as indicated on each image.


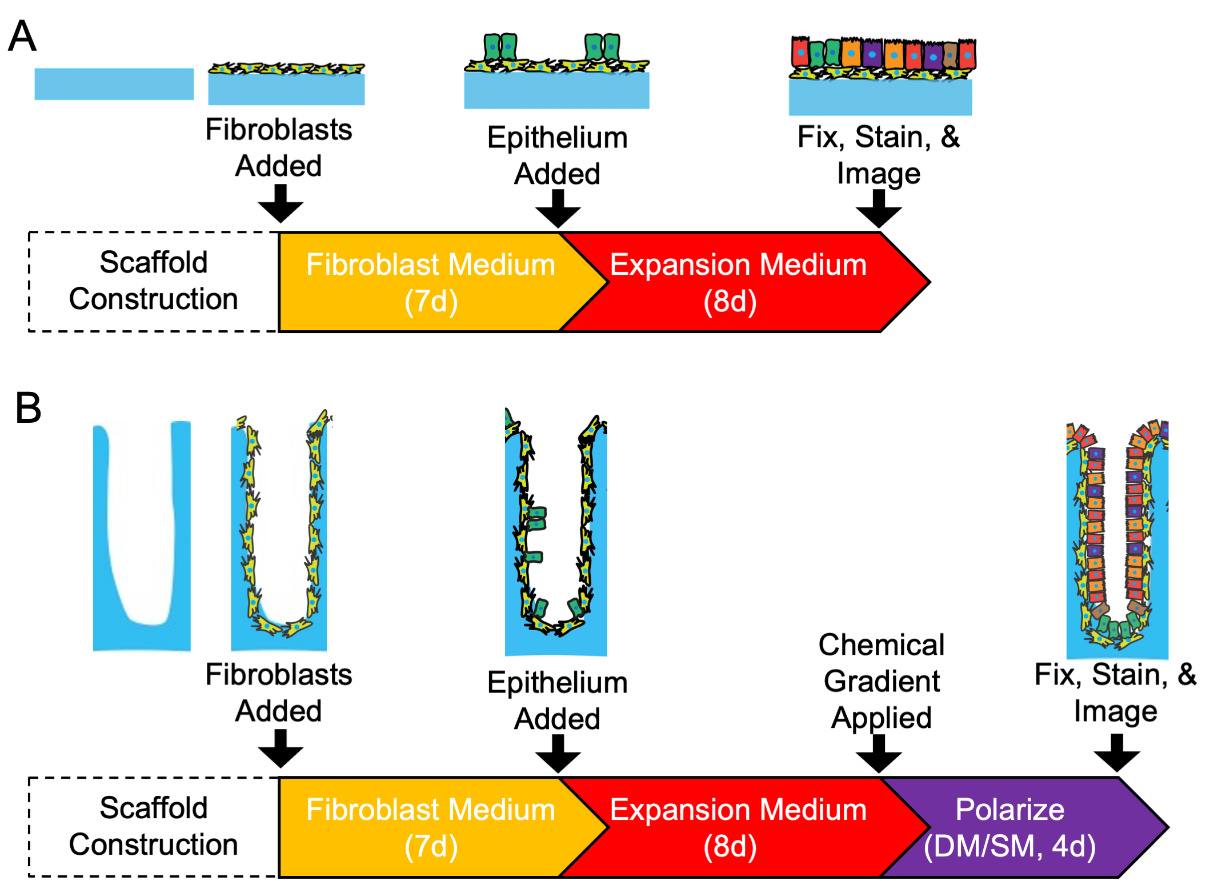


**Supplemental Figure S2.** Timeline for in vitro tissue seeding. (A) Planar model timeline: Fibroblasts are added first atop a cross-linked collagen scaffold and cultured in FM for one week after which epithelium is added and the full tissue is culture in EM. (B) 3D-crypt model timeline: Like the planar setup, fibroblasts are first added to the scaffold and culture for one week in FM followed by epithelium addition. Cells are cultured in EM (supplied from above and below the scaffold) for eight days and then a chemical gradient is applied with DM supplied at the top of the scaffold within a well-insert and SM is supplied at bottom from below the well-insert. In all cases 0.5 mL of medium is added to the top of the well-insert and 1.5 mL is added below the well insert and the medium is replenished every 24 hours.


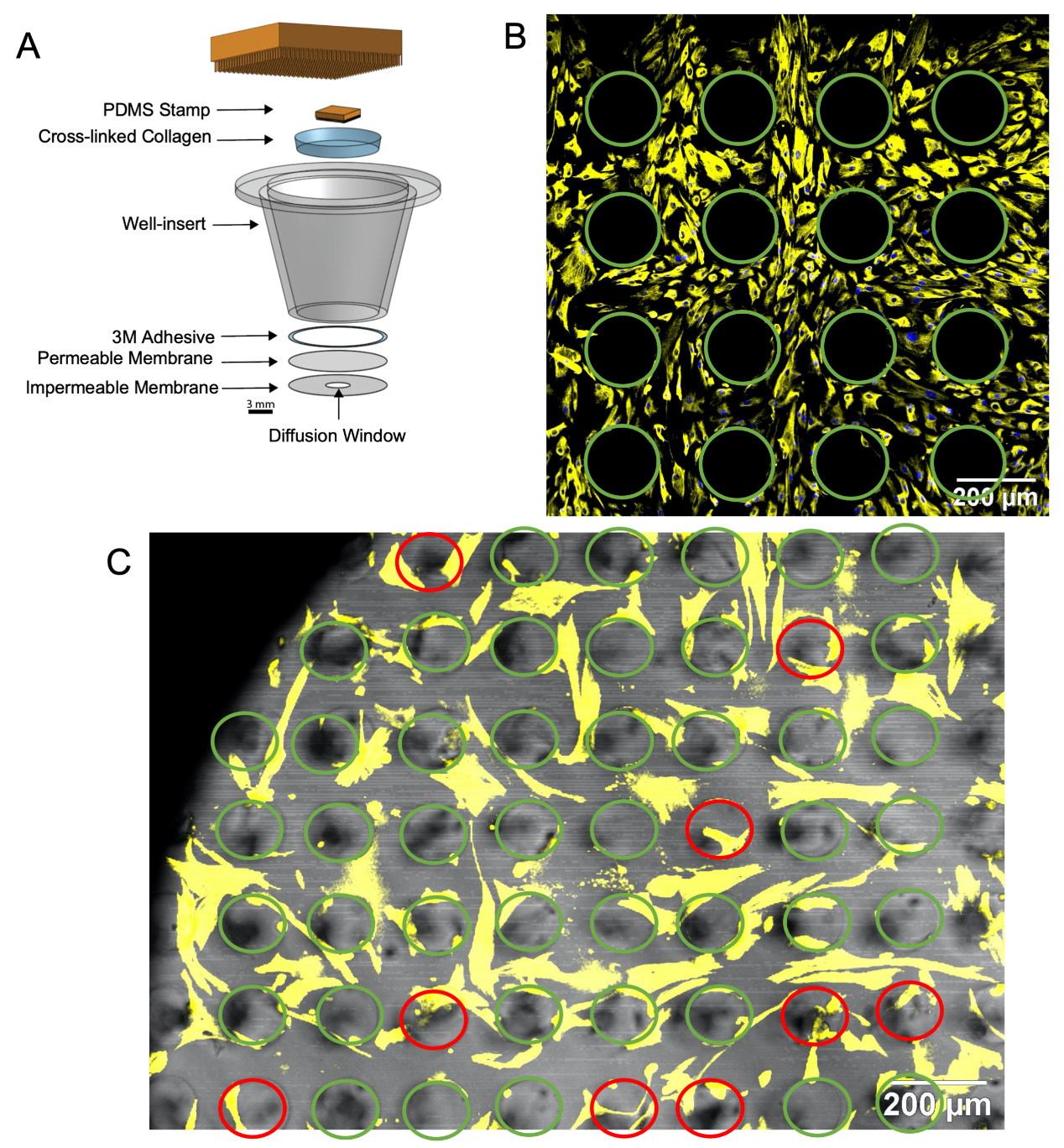


**Supplemental Figure S3.** Expanding the size of crypts to accommodate fibroblasts. (A) An exploded view of the components within modified well-inserts with a 3 mm diffusion window that are used in both the planar and 3D-crypt models. (B) Immunofluorescence image displaying fibroblasts on a scaffold with 150 µm-wide crypts (yellow = Vimentin, blue = Hoechst 33342). The focal plane for this image is at the top of the scaffold near the crypt openings and it shows that 100 % of crypts with a wider 150 µm opening are not blocked by spanning fibroblasts. (C) Immunofluorescence image displaying fibroblasts atop a scaffold with 80 µm-wide crypts (yellow = Vimentin). Red circles over an opening show a crypt that is occluded by fibroblasts into which epithelial cells could not be added and green circles denote clear opening (9/53 crypts occluded, 17%).


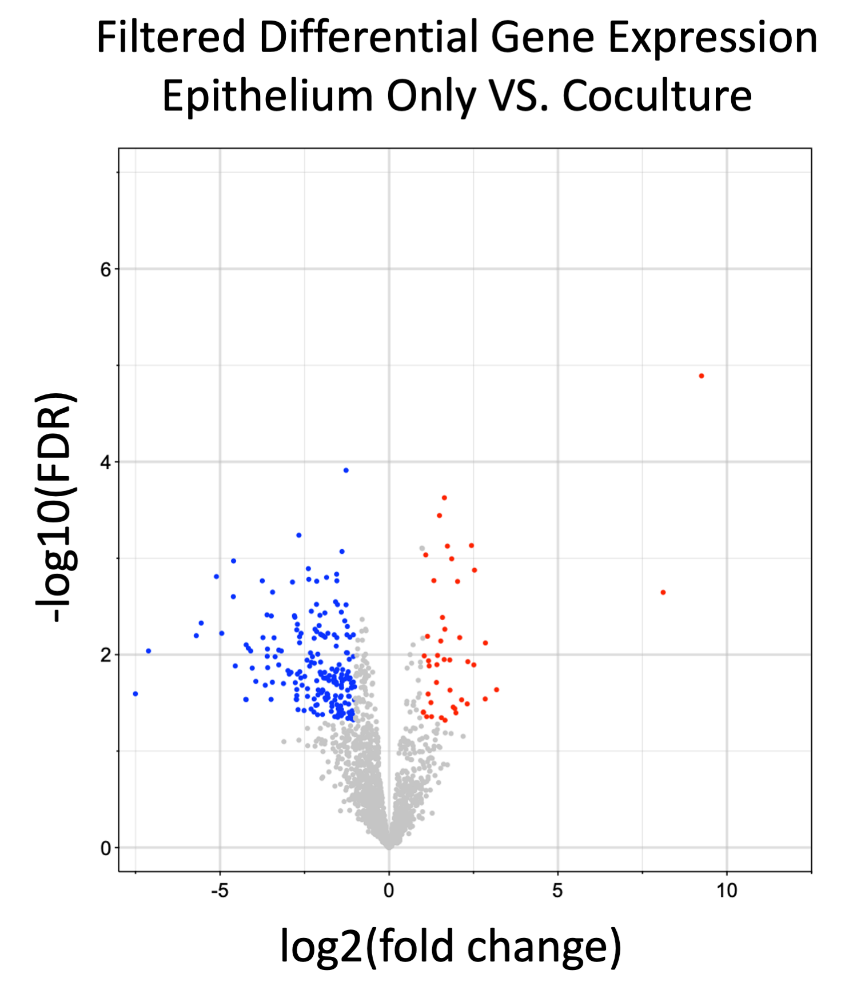


**Supplemental Figure S4.** Differential gene expression for genes not expressed by fibroblasts. Gray dots display genes that are not significantly different between epithelium only and coculture samples while red dots signify significantly upregulated genes and blue dots show significantly downregulated genes in coculture. N.b., genes with significantly different expression are listed in Supplemental Table S4 and S5.

**References**

Hinman, S. S. *et al.* (2021) ‘In vitro generation of self-renewing human intestinal epithelia over planar and shaped collagen hydrogels’, *Nature Protocols*. Nature Research, 16(1), pp. 352–382. doi: 10.1038/s41596-020-00419-8.
